# Supplementary material for: Hydride-based antiperovskites with soft anionic sublattices as fast alkali ionic conductors
Source: Nat Commun. 2021 Jan 8;12:201. doi: 10.1038/s41467-020-20370-2 (PMC7794446; doi:10.1038/s41467-020-20370-2)
Supplement: Supplementary file 1 — Supplementary Information [file 41467_2020_20370_MOESM1_ESM.pdf]

Supplementary Information

Hydride-based antiperovskites with soft anionic sublattices as fast alkali ionic conductors

Shenghan Gao,[a,‡] Thibault Broux,[a,‡] Susumu Fujii,[b,‡] Ce dric Tassel,[a,*] Kentaro Yamamoto,[c] Yao Xiao,[c] Itaru Oikawa,[d] Hitoshi Takamura,[d] Hiroki Ubukata,[a] Yuki Watanabe,[a] Kotaro Fujii,[e] Masatomo Yashima,[e] Akihide Kuwabara,[b] Yoshiharu Uchimoto,[c] and Hiroshi Kageyama[a,*]

[a]*Department of Energy and Hydrocarbon Chemistry, Graduate School of Engineering, Kyoto University, Nishikyo-ku, Kyoto 615-8510, Japan*

[b]*Nanostructures Research Laboratory, Japan Fine Ceramics Center, Nagoya 456-8587, Japan*

[c]*Graduate School of Human and Environmental Studies, Kyoto University, Sakyo-ku, Kyoto 606-8501, Japan*

[d]*Department of Materials Science, Graduate School of Engineering, Tohoku University, Sendai 980-8579, Japan*

[e]*Department of Chemistry, School of Science, Tokyo Institute of Technology, 2-12-1-W4-17 O-okayama, Meguro-ku, Tokyo 152-8551, Japan*

Table of Contents: Page

Section 1. Supplementary Methods -- S3-S4
Section 2. Crystal structure analysis of M_3HCh antiperovskites ---------------------- S5-S14
Supplementary Figures 1-9
Supplementary Tables 1-5
Section 3. Size-flexibility of hydride anion --- S15-S18
Supplementary Figure 10-11
Supplementary Table 6-7
Section 4. Ambient synthesis of Na_3HTe -- S19
Supplementary Figure 12
Supplementary Table 8
Section 5. Electronic band structures at 0 GPa--S20
Supplementary Figure 13
Section 6. Phonon band structures at 0 and 5 GPa -------------------------------------- S21-S23
Supplementary Figure 14
Section 7. Na^+ migration pathways in orthorhombic Na_3HS---------------------------------S24
Supplementary Figure 15
Section 8. The migration barriers and defect formation energies in M_3HCh -------------S25
Supplementary Figure 16
Supplementary Table 9
Section 9. Ionic diffusion -- S26-S30
Supplementary Figures 17-21
Supplementary Table 10
Section 10. Supplementary references --S31

Section 1. Supplementary Methods

Theoretical Calculations. Total energy calculations and structural optimizations of M_3HCh, MH and M_2Ch under 0 and 5 GPa were performed with Vienna *Ab initio* Simulation Package (VASP) (Kresse, G. & Hafner, J. *Phys. Rev. B* **1993**, 47, 558–561; Kresse, G. & Furthmu ller, J. *Phys. Rev. B* **1996,** 54, 11169–11186), using the plane-wave basis projector augmented wave (PAW) method (Blöchl, P. E. *Phys. Rev. B* **1994,** 50, 17953–17979) and the generalized gradient approximation (GGA) in the form of PBEsol (Perdew, J. P. *et al. Phys. Rev. Lett.* **2008,** 100, 136406). We used PAW potentials with the following valence configurations: $1s^22s^1$ for Li, $2p^63s^1$ for Na, $1s^1$ for H, $3s^23p^4$ for S, $4s^24p^4$ for Se and $5s^25p^4$ for Te. An energy cutoff of 650 eV was used for the plane-wave basis set. The criteria of convergence for electronic self-consistent cycle and structure optimization were set to be 1.0×10^{-6} eV/atom and 1.0×10^{-3} eV/A , respectively. The Brillouin zones were sampled with Γ-centered k-point meshes with the spacing of 0.2 A $^{-1}$ (and 0.1 A $^{-1}$ for the calculations of electronic density of states). Enthalpies of M_3HCh, MH and M_2Ch were calculated according to the following equation:

$$H(p) = U(V) + pV \quad (1),$$

where H, U, p and V are enthalpy, internal energy, pressure and volume.

Phonon dispersions and vibrational free energies were calculated using the Parlinsk-Li-Kawazoe method (Parlinski, K., Li, Z. Q. & Kawazoe, Y. *Phys. Rev. Lett.* **1997,** 78, 4063–4066) as implemented in Phonopy (Togo, A. & Tanaka, I. *Scr. Mater.* **2015,** 108, 1–5). Dynamical matrixes were constructed from real space interatomic force constants. To determine the force constants, symmetrically nonequivalent atomic displacements with the distance of 0.01 A were introduced to the stationary atomic positions. For these phonon calculations, 2 × 2 × 2 supercells were used for all compounds after the preliminary tests using 3 × 3 × 3 and 4 × 4 × 4 supercells of Li_3HS. Vibrational free energies, F_{vib}, were calculated with dense q-point meshes containing at least 560 q-points. Gibbs free energies of M_3HCh, MH and M_2Ch were calculated as below:

$$G(T, p) = U(V) + pV + {}_{vjb(T,}V) = H(p) + {}_{vjb(T,}V) \quad (2),$$

where G and T are Gibbs free energy and temperature. In the present study, only vibrational entropy was considered, not configurational entropy. Phonon band centers for M_+ ions were calculated as the average phonon frequencies, W_{ave}, by the following equation: $f\, W.\, g(W)\, dW$

$$W_{ave} = \frac{\quad}{f g(W) dW} \quad (3),$$

where W and g are phonon frequency and phonon density of states (DOS). Imaginary frequencies were neglected for the calculations.

The force constant k_{rot} with respect to the phonon mode at M point, which corresponds to the rotational motion of a HM_6 octahedron, was estimated by $k_{rot} = W_{rot}^2 . m_M$, where W_{rot} and m_M are the frequency of the rotational phonon mode and the atomic mass of M, respectively. This is in accordance with the equation of simple harmonic motion $W =$

$\sqrt{k/m}$.

The Bader population analysis, which partitions a charge density grid into the Bader region of each ion, was performed to investigate the size variation of each ion upon changing M and Ch species and applying external pressure (Tang, W., Sanville, E. & Henkelman, G. *J. Phys. Condens. Matter* **2009**, 21, 084204). The partition results for cubic Li_3HS are shown in Supplementary Figure 11 as an example. The volume of the partitioned region (Bader volume) was used to estimate the radius of each ion (Bader radius) according to the equation ${}_B = \frac{4}{3}\ {}_B^3$, where V_B and r_B are the Bader volume and radius, respectively (Harashima, Y., Terakura, K., Kino, H., Ishibashi, S. & Miyake, T. *J. Appl. Phys.* **2016**, 120, 203904). Note that the Bader radii are larger than the commonly used ionic radii such as Shannon ionic radii since Bader regions are space filling whereas spheres are not.

The nudged elastic band (NEB) calculations (Mills, G., Jónsson, H. & Schenter, G. K. *Surf. Sci.* **1995,** 324, 305–337) were performed to estimate energy barriers for ion migration mediated by vacancy or interstitial defects. The climbing image NEB method (Henkelman, G., Uberuaga, B. P. & Jónsson, H. *J. Chem. Phys.* **2000,** 113, 9901–9904) was employed for orthorhombic Na_3HS. For these calculations, 3 × 3 × 3 or 2 × 2 × 2 charged supercells were used for cubic M_3HCh and orthorhombic Na_3HS, respectively. The Brillouin zones of the supercells were sampled with one *k*-point at (0.25 0.25 0.25). The criteria of convergence for electronic self-consistent cycle and structure optimization in NEB calculations were set to be 1.0×10^{-6} eV/supercell and 2.0×10^{-2} eV/A , respectively. The total energies of the defect models calculated with the same condition were used to estimate the formation energies of Schottky and Frenkel defects.

Section 2. Crystal structure analysis of M_3HCh antiperovskites

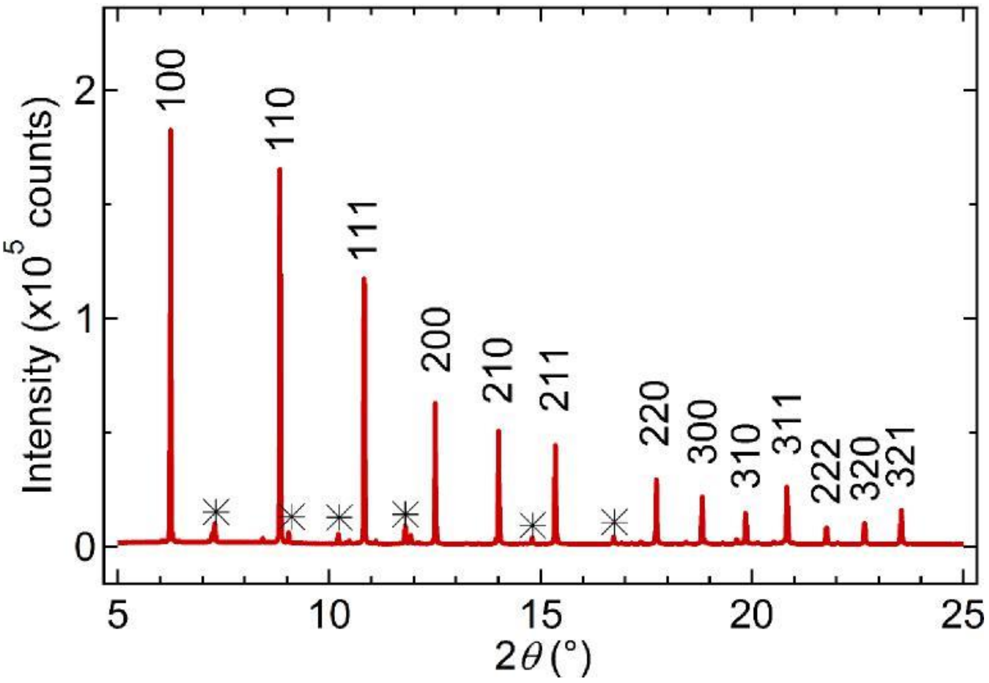

Supplementary Figure 1 SXRD pattern of Li_3HS synthesized from a mixture of Li_2S and LiH (molar ratio of 1:3) at 5 GPa and 700 °C. The diffraction peaks are indexed in a cubic symmetry and labelled with the Miller indices of the lattice planes. Asterisks denote unreacted precursor impurities.

Supplementary Table 1 Atomic coordinates, displacement parameters and occupancy factors for the cubic *3*[illegible] structure of Li_3HS by SXRD (upper)[a] /ND (lower).[b]

Atom	Site	x	y	z	U_{iso} U_{ii} (100 Å^2)	*g*
Li	3*d*	0.5	0	0	U_{11} = 1.73(16) 0.11(8) U_{22} = U_{33} = 4.41(14) 3.79(10)	0.98(7)/1
S	1*b*	0.5	0.5	0.5	1.05(12) 0.43(4)	1
H	1*a*	0	0	0	0.63* 1.61(5)	1

[a]For SXRD (λ = 0. 0.41989 A) refinement using FULLPROF program, *a* = 3.85189(6) A . R_{Bragg} = 4.28% and R_f = 2.87%, 78.1vol% Li_3HS with the impurity of 0.3vol% Li_2S, 17.7vol% LiH, and 3.9vol% BN.

[b]For ND refinement, *a* = 3.853354(17) A , R_{wp} = 1.35% and R_{Bragg} = 6.28%, weight fraction of 67.06%. Rietveld refinement assuming the anion exchange between *A* and *B* site (g_A = g_A(S) + g_A(H), g_B = g_B(S) + g_B(H), g_A(H) + g_B(H) = 1, and g_A(S) + g_B(S) = 1) yield R_{wp} = 1.37% and R_{Bragg} = 6.64% with the occupancies of g_B(H) = 1.015(5) and g_A(H) = –0.015(5).

*The isotropic displacement parameter of H^- was not refined for SXRD.

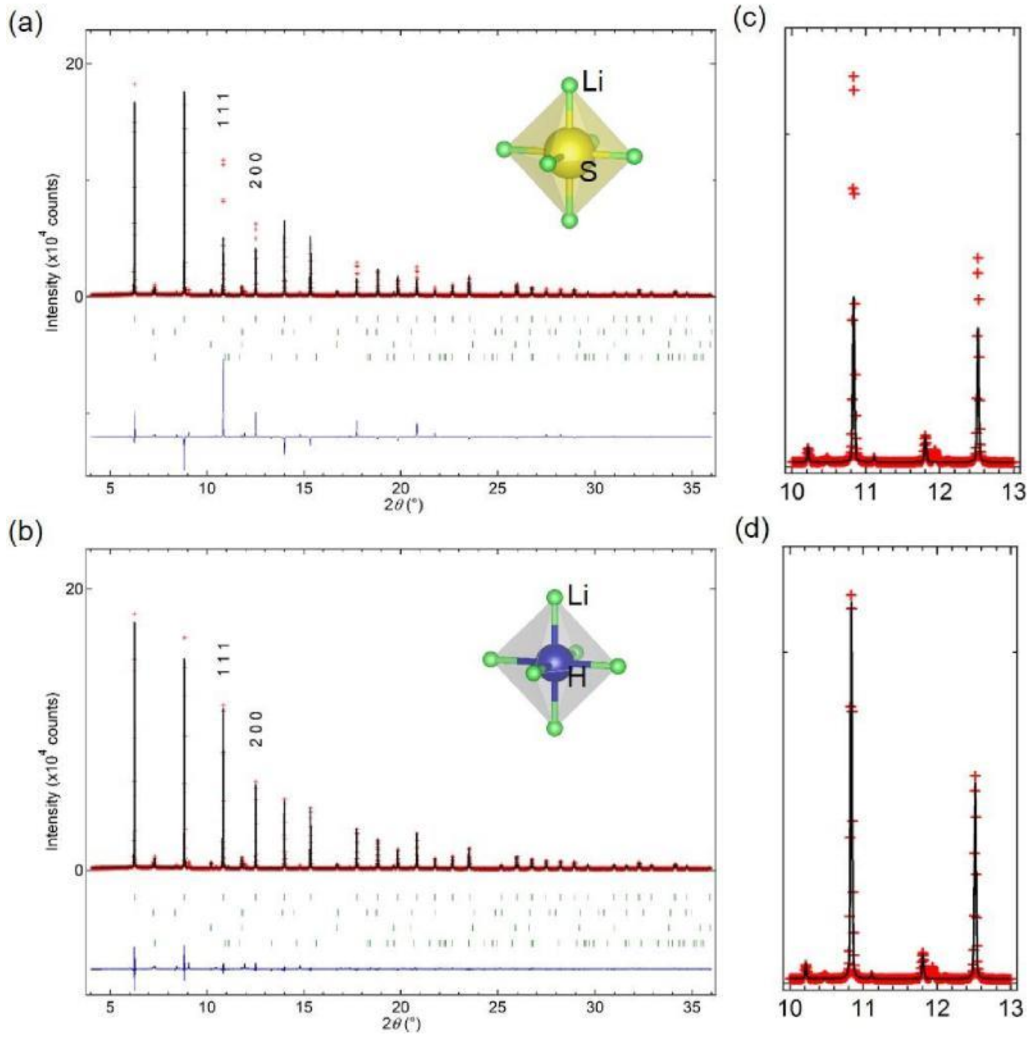

Supplementary Figure 2 Rietveld refinement of SXRD data of *3* Li_3HS using two different anion arrangements with S^{2-} centered in a Li octahedron (a, c) and H^- centered in a Li octahedron (b, d). The right panel of magnified reflections of 111 and 200 compares the intensities of the observed (red crosses) and calculated (black solid line) patterns under two proposed anion distributions. The red crosses, black solid line, the blue solid line, and green dashes denote the observed, calculated, difference intensities and calculated Bragg reflections (from top to bottom: Li_3HS, Li_2S, LiH, BN), respectively.

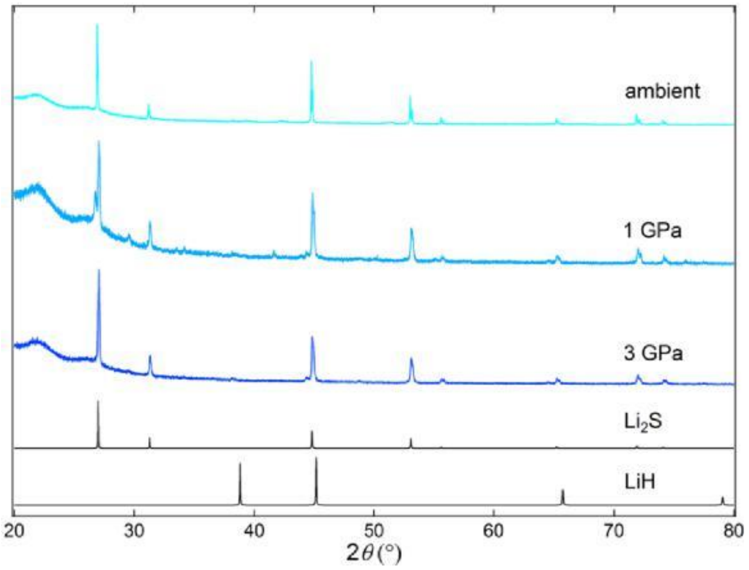

Supplementary Figure 3 The diffraction patterns of obtained powders from reactions of mixtures of Li_2S and LiH under ambient pressure, 1 GPa and 3 GPa at 700 °C. The broad hump in the low-angle range originates from the Kapton tape used to cover the sample and in order to avoid the moisture and air contamination during the measurement.

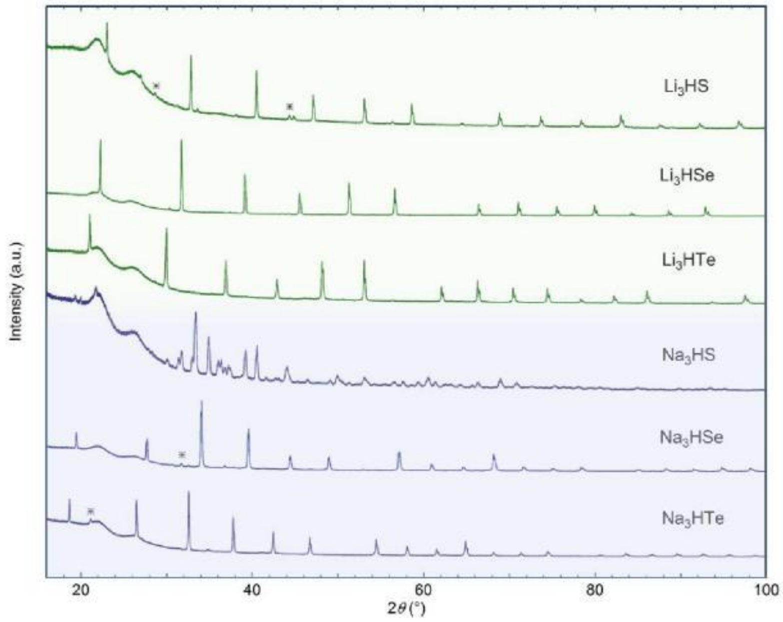

Supplementary Figure 4 All the diffraction patterns of the new class of M_3HCh antiperovskites (M = Li, Na; Ch = S, Se, Te). All data were collected from a laboratory X-ray diffractometer with Cu-Kα radiation, where the broad hump in the low-angle range originates from the Kapton tape used to cover the sample powders to avoid the moisture and air contamination during the measurement. Asterisks denote a few minor impurity peaks.

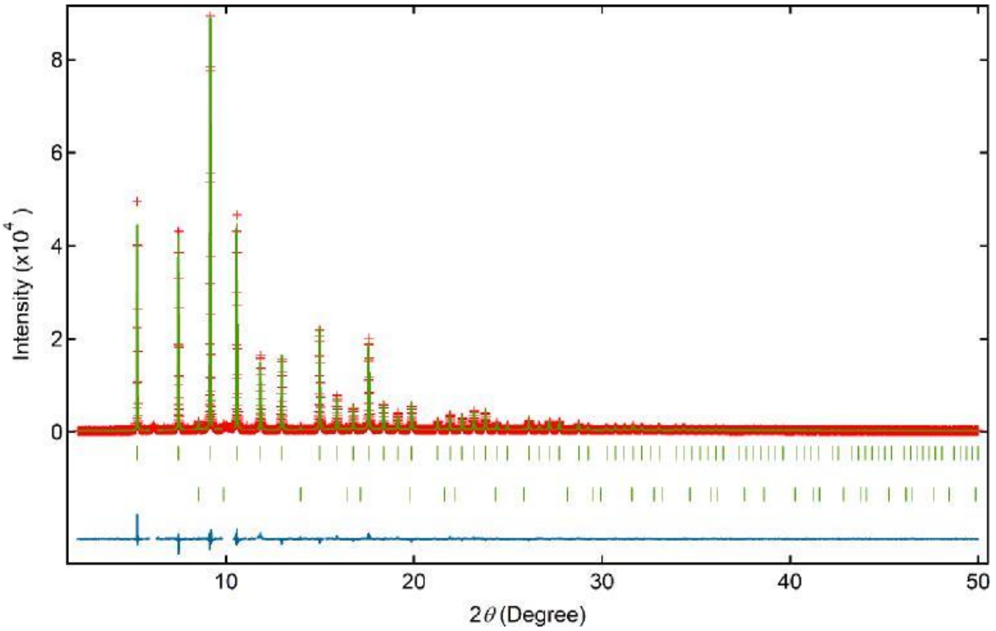

Supplementary Figure 5 Rietveld refinement of SXRD (λ = 0.42043 A) pattern of Na_3HSe. The red crosses, green solid line, the blue solid line, and green dashes denote the observed, calculated, difference intensities and calculated Bragg reflections (from top to bottom: Na_3HSe, NaH), respectively. A few broad peaks of Na_2Se and unknown impurity can be observed in the following ranges and were excluded from the refinement: 4.941–4.985°, 5.947–6.313°, 5.656–5.687°, 6.681–6.741°, 7.938–7.985°, 9.322–9.385°, 9.824–10.423°, 10.363–10.41°, 10.815–10.862°, 11.062–11.138°, 11.583–11.671°, 15.239–15.286°, and 15.559–15.622°.

Supplementary Table 2 Atomic coordinates, displacement parameters and occupancy factors for the cubic Na_3HSe by SXRD[a]

Atom	Site	x	y	z	U_{iso} U_{ii} (100 Å^2)	g
Na	3*d*	0.5	0	0	U_{11} = 1.01(7)	0.996(2)
Se	1*b*	0.5	0.5	0.5	1.708(12 = 515(6)	1
H	1*a*	0	0	0	1*	1

[a]Space group *3*[illegible] (No. 221), *a* = 4.55901(7) Å. R_{wp} = 7.16%, GOF = 2.04, R_{Bragg} = 4.99% and R_f =3.97% for SXRD using JANA2006 program. Mass fractions are Na_3HSe = 96.3(3)%, NaH = 3.71(7)%. Rietveld refinement assuming the anion exchange between *A* and *B* site ($g_A = g_A$(Se) + g_A(H), $g_B = g_B$(Se) + g_B(H), g_A(H) + g_B(H) = 1, and g_A(Se) + g_B(Se) = 1) yielded R_{wp} = 6.60% and R_{Bragg} = 2.80% with the occupancies of g_A(Se) = 1.0236(7) and g_A(H) = – 0.0236(7). The refined composition is $Na_{2.988(9)}HSe$.

*The isotropic displacement parameter is not refined.

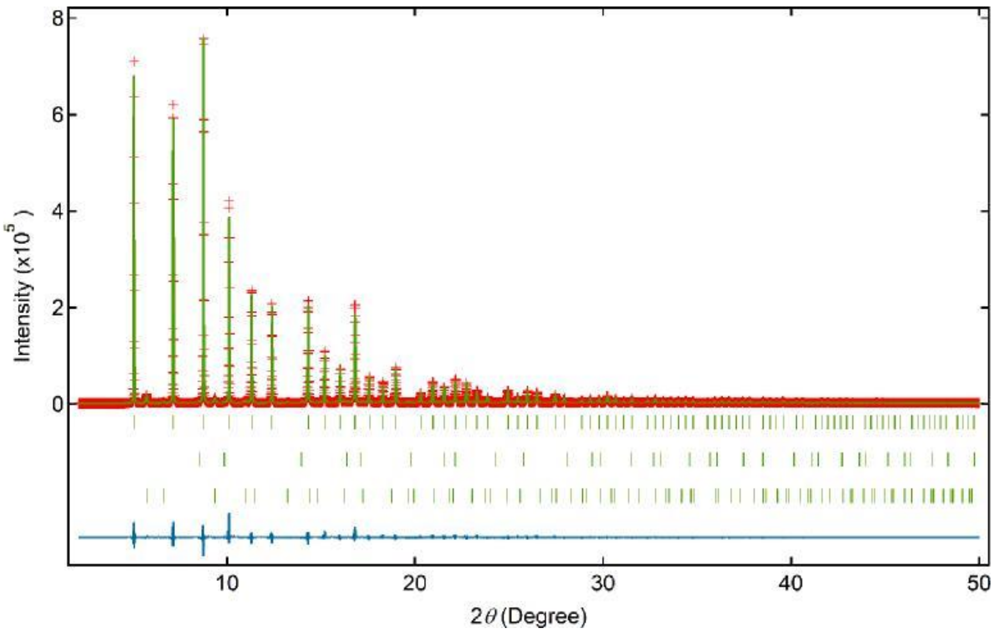

Supplementary Figure 6 Rietveld refinement of SXRD (λ = 0.41989 A) pattern of Na_3HTe. The red crosses, green solid line, the blue solid line, and green dashes denote the observed, calculated, difference intensities and calculated Bragg reflections (from top to bottom: Na_3HTe, NaH, Na_2Te), respectively.

Supplementary Table 3 Atomic coordinates, displacement parameters and occupancy factors for the cubic Na_3HTe by SXRD[a]

Atom	Site	x	y	z	U_{iso} U_{ii} (100 Å^2)	*g*
Na	3*d*	0.5	0	0	U_{11} = 1.65(9) 1.551(9) = 3 106	0.986(3)
Te	1*b*	0.5	0.5	0.5		1
H	1*a*	0	0	0	1*	1

[a]Space group 3[illegible] (No. 221), *a* = 4.76349(2) Å. R_{wp} = 7.72%, GOP = 6.38, R_{Bragg} = 3.71% and R_f = 2.53% for SXRD using JANA2006 program. Mass fractions are Na_3HTe = 95.3(3)%, NaH = 1.3198(3)%, Na_2Te = 3.37(3)%. Rietveld refinement assuming the anion exchange between *A* and *B* site ($g_A = g_A$(Te) + g_A(H), $g_B = g_B$(Te) + g_B(H), g_A(H) + g_B(H) = 1, and g_A(Te) + g_B(Te) = 1) yielded R_{wp} = 7.68% with the occupancies of g_A(Te) = 0.9828(6) and g_A(H) = 0.0172(6). The refined composition is $Na_{2.958(9)}HTe$.

*The isotropic displacement parameter is not refined.

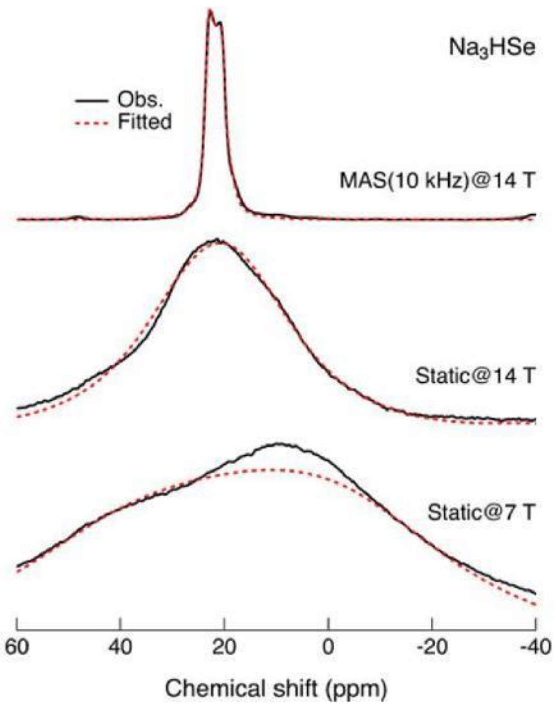

Supplementary Figure 7 Magnetic field dependence of ^{23}Na NMR spectra of Na_3HSe at room temperature (from bottom to top, under a magnetic field of 7 T, 14 T, and 14 T with magic-angle spinning at 10 kHz). All the three spectra were fitted as a 2nd-order quadrupolar line shape with a common set of C_Q = 1.61 MHz and ŋ = 0.04, where C_Q, and ŋ denote a quadrupole coupling constant and an asymmetry parameter, respectively.

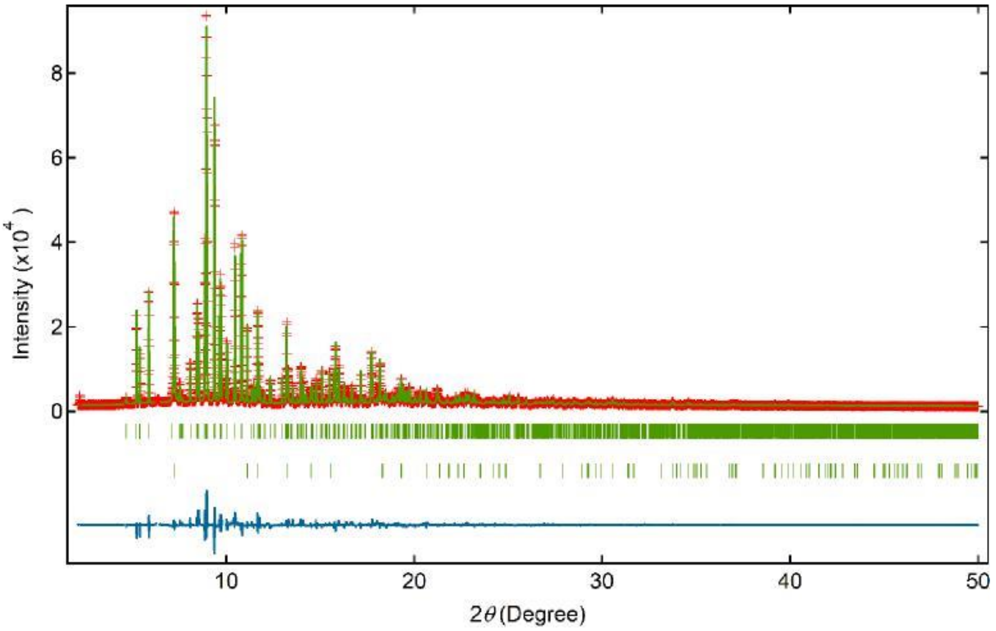

Supplementary Figure 8 Rietveld refinement of SXRD (λ = 0.41989 A) pattern of Na_3HS. The red crosses, green solid line, the blue solid line, and green dashes denote the observed, calculated, difference intensities and calculated Bragg reflections (from top to bottom: Na_3HS, h-BN), respectively. The following ranges were excluded from the refinement: 2.168–2.264°, 4.573–4.659°, 6.167–6.227°, 6.298–6.441°, 9.806–9.996°, 16.927–17.01° , and 50-71.439°

Supplementary Table 4 Atomic coordinates, displacement parameters and occupancy factors for the orthorhombic Na_3HS by SXRD[a]

Atom	Site	x	y	z	U_{iso} U_{ii} (100 Å2	*g*
Na1	4*c*	0.0331(2)	0.25	0.5932(2)	U_{11} = 1.84(11)	1
Na2	8*d*	0.19451(15)	0.04941(8)	0.19206(16)	U_{11} = 2.81(8 U_{22} = 1 77(10)	1
S	4*c*	0.43047(15)	0.25	0.48481(16)	[illegible] U_{33} = 2 35(12	1
H	4*b*	0.5	0.5	0	[illegible]* 8)	1

[a]Space group (No. 62), *a* = 6.76037(9)Å, *b* = 8.89761(10)Å, *c* = 6.8659(8)Å. R_{wp} = 6.13%, GOF = 3.09, R_{Bragg} = 7.58% and R_f =5.99% for SXRD using JANA206 program. The h-BN peaks were refined using Le Bail analysis. When the occupancies of the Na1 and Na2 site were refined a composition of $Na_{3.129(9)}HS$ was obtained with R_{wp} = 6.02%, *g*(Na1) = 1.039(3) and *g*(Na2) = 1.045(3). Rietveld refinement assuming the anion exchange between *A* and *B* site ($g_A = g_A(S) + g_A(H)$, $g_B = g_B(S) + g_B(H)$, $g_A(H) + g_B(H) = 1$, and $g_A(S) + g_B(S) = 1$) yielded R_{wp} = 5.93% and R_{Bragg} =7.07% with the occupancies of $g_A(S)$ = 0.9781(10) and $g_A(H)$ = 0.0219(10

U = 034(7) [illegible]
U_{13} = 046(7)
U_{23} = 081(6)

*The isotropic displacement parameter is not refined.

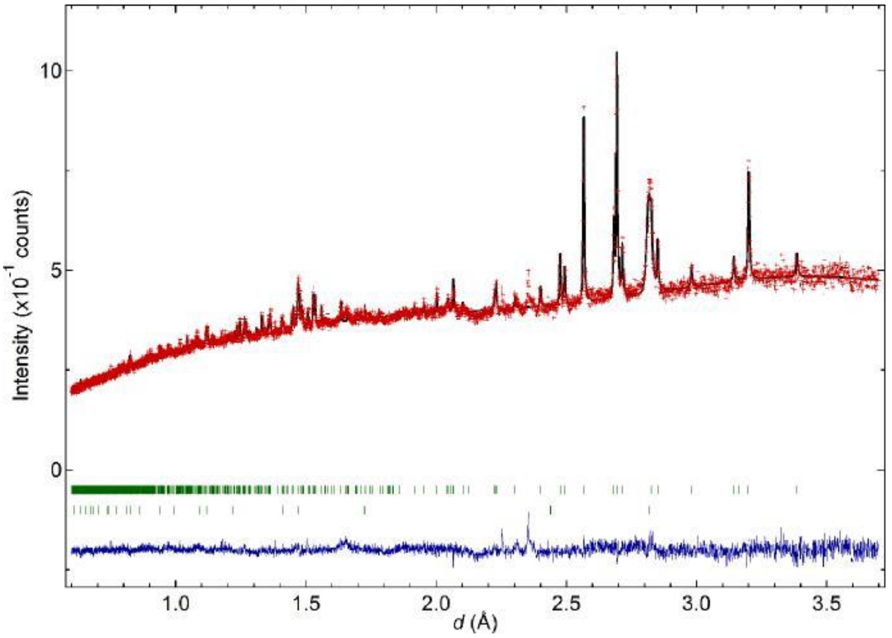

Supplementary Figure 9 Rietveld refinement of PND pattern of Na_3HS. The red crosses, black solid line, the blue solid line, and green dashes denote the observed, calculated, difference intensities and calculated Bragg reflections (from top to bottom: Na_3HS, NaH), respectively.

Supplementary Table 5 Atomic coordinates, displacement parameters and occupancy factors for the orthorhombic Na_3HS by PND[a]

Atom	Site	x	y	z	U_{iso} U_{ii} (100 Å^2)	*g*
Na1	4*c*	0.03795(9)	0.25	0.59096(9)	U_{11} = 1.90(4)	1
Na2	8*d*	0.19253(5)	0.04994(5)	0.1906(6)	U_{11} = 1.68(2) [illegible] 2.274 [illegible] U_{12} = 0.53(2) U_{13} = 0.68(2)	1
S	4*c*	0.42885(9)	0.25	0.48863(11)	[illegible] 2.344	1
H	4*b*	0.5	0.5	0	[illegible]	1

[a]Space group (No. 62), *a* = 6.77242(12) Å, *b* = 8.89811(2) Å, *c* = 6.28671(12) Å. R_{wp} = 2.53% and R_{Bragg} = 24.30%, weight fraction = 89.72% for PND using Z-Rietveld program.

Section 3. Size-flexibility of hydride anion

Supplementary Table 6 Information of M_3HCh (M = Li, Na; Ch = S, Se, Te).

Composition	Space group	Lattice parameters (Å)	Ionic radius (ppm) [a]		t [b] / t'	Ch^{2-} BVS [e]
			Li	76		
Li_3HS	Pm3 m	a = 3.85189(6)	S	184		−1.44
			H	140	0.85 [c]	
				116.6	0.95 [d]	
			Li	76		
Li_3HSe	Pm3 m	a = 3.9744(5)	Se	198		−1.72
			H	140	0.90	
				122.6	0.98	
			Li	76		
Li_3HTe	Pm3 m	a = 4.2221(3)	Te	221		−1.87
			H	140	0.97	
				135.3	0.99	
			Na	102		
Na_3HS	Pnma	a = 6.76037(9)<br>b = 8.89761(10)<br>c = 6.28659(8)	S	184		−1.60
			H	140	0.84	
				135.5	0.85	
			Na	102		
Na_3HSe	Pm3 m	a = 4.55901(7)	Se	198		−1.32
			H	140	0.88	
				126.2	0.93	
			Na	102		
Na_3HTe	Pm3 m	a = 4.76349(2)	Te	221		−1.67
			H	140	0.94	
				136.3	0.96	

[a] The ionic radii for r_{Ch}, r_M are referred as revised effective Shannon ionic radii (Shannon, R. D. *Acta Cryst.* **1976**, *32*, 751). It should be noted that Shannon ionic radius of r_{Ch} for coordination number (CN) six was used as a rough estimation, though the CN of Ch in M_3HCh antiperovskites is twelve. The value of r_H = 140 pm is employed in many oxides and pure hydrides (Messer C. E. *J. Solid State Chem.* **1970**, 2, 144).

[b] The Goldschmidt tolerance factor of M_3HCh (M = Li, Na; Ch = S, Se, Te) is defined by the equation $t = (r_{Ch} + r_M)/[\sqrt{2}(r_H + r_M)]$, where r_{Ch}, r_M and r_H are the ionic radius of chalcogenide, alkali and hydride ion, respectively.

[c] The switching anion configuration from Li_3HS (HLi_6 octahedra) to Li_3SH (SLi_6 octahedra) led to a very low t value of 0.59. By comparison with t value of Li_3HS (0.85), the smaller t indicates the higher instability and a tendency to decomposition.

[d] The refined tolerance factor (t') for M_3HCh using the observed ionic radius of hydride.

[e] We calculated bond valence sum (BVS) parameters of Ch^{2-} based on the reported Ch-Li/Na according to the reference (Brese, N. E.; O'Keeffe, M. *Acta Cryst.* **1991**, *47*, 192).

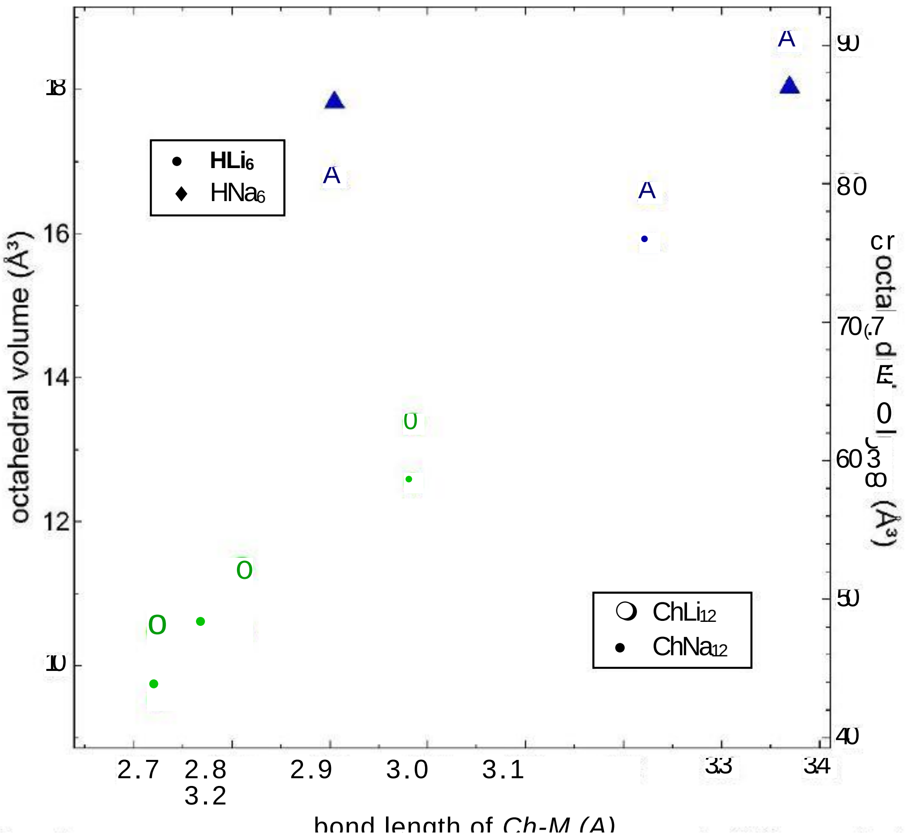

Supplementary Figure 10 The volume change of HM_6 octahedra and ChM_{12} cuboctahedra in the M_3HCh antiperovskites

Supplementary Table 7 Bader radius of M_+, H^- and Ch^{2-} ions in M_3HCh compounds (M = Li, Na; Ch = S, Se, Te) under the pressure of 0 and 5 GPa.

	Pressure (GPa)	Bader Atomic Radius (Å)			Normalized Bader Atomic Radius		
		M	H	Ch	M	H	Ch
Li_3HS	0	0.938	1.377	1.968	1.000	1.000	1.000
	5	0.920	1.326	1.908	0.980	0.963	0.970
Li_3HSe	0	0.952	1.421	2.057	1.000	1.000	1.000
	5	0.931	1.362	1.991	0.978	0.958	0.968
Li_3HTe	0	0.972	1.482	2.223	1.000	1.000	1.000
	5	0.948	1.415	2.142	0.975	0.955	0.963
c-Na_3HS	0	1.322	1.477	2.151	1.000	1.000	1.000
	5	1.281	1.388	2.060	0.969	0.940	0.958
Na_3HSe	0	1.332	1.515	2.235	1.000	1.000	1.000
	5	1.288	1.428	2.132	0.967	0.943	0.954
Na_3HTe	0	1.350	1.574	2.386	1.000	1.000	1.000
	5	1.300	1.479	2.269	0.963	0.939	0.951

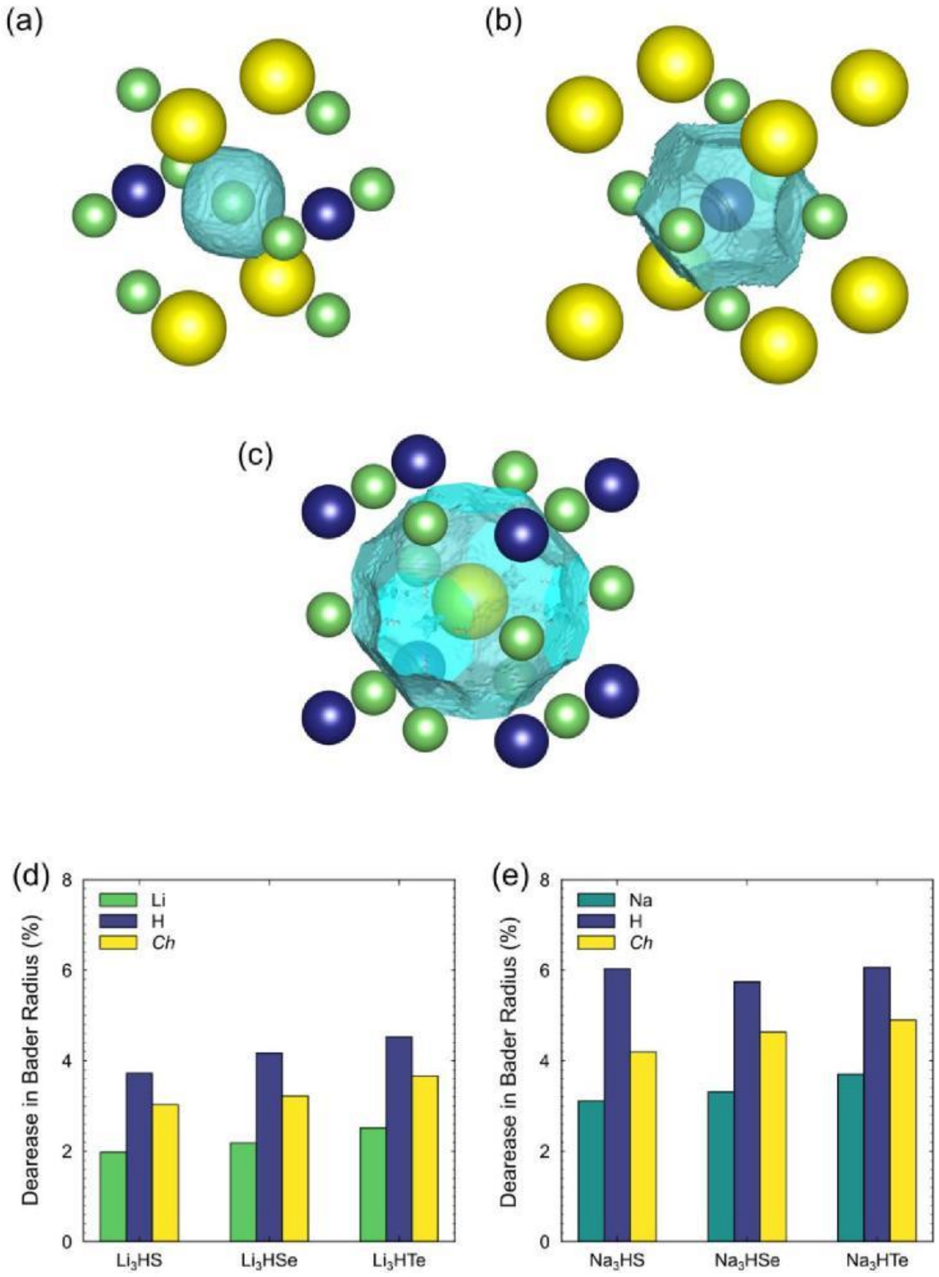

Supplementary Figure 11 Bader region (light blue) of each ion (a) Li^{+} (green), (b) H^{-} (blue) and (c) S^{2-} (yellow) in Li_3HS. Decrease percentage of Bader radius of each ion in (d) Li_3HCh and (e) Na_3HCh under the pressure of 5 GPa.

Section 4. Ambient synthesis of Na_3HTe

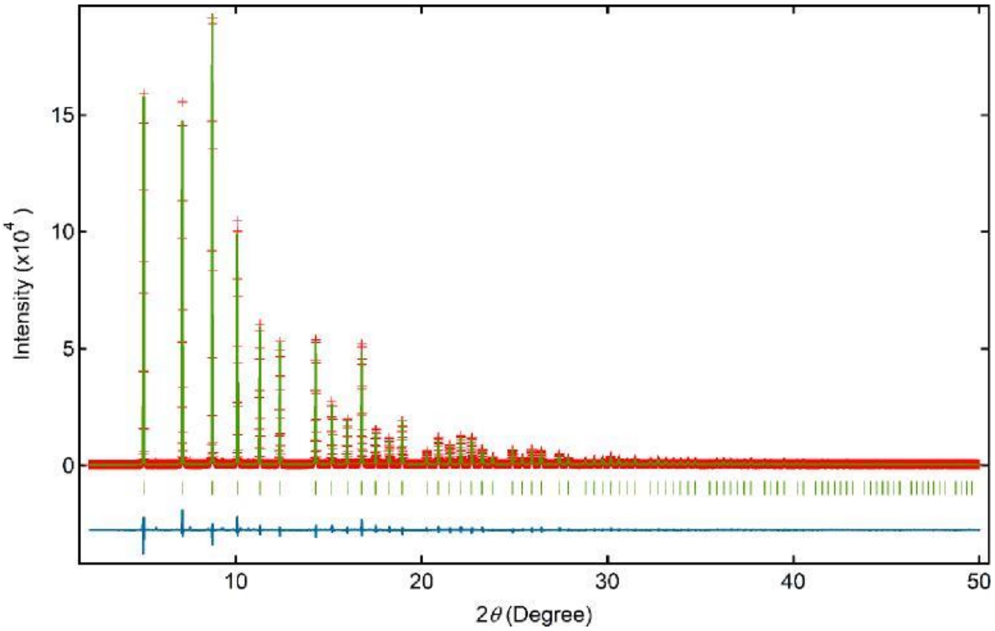

Supplementary Figure 12 Rietveld refinement of SXRD (λ = 0.41943 A) pattern of Na_3HTe obtained at ambient pressure. Na_2Te (Kojundo, 99%) was mixed with NaH (Sigma-Aldrich, 99%) in a 1:2 molar ratio, sealed in an evacuated pyrex tube, and heat at 400 °C for 12 h. The red crosses, green solid line, the blue solid line, and green dashes denotes the observed, calculated, difference intensities and calculated Bragg reflections, respectively. The refinement was performed between 2º and 50º.

Supplementary Table 8 Atomic coordinates, displacement parameters and occupancy factors for Na_3HTe ambient phase by SXRD[a]

Atom	Site	x	y	z	U_{iso} / U_{ii} (100 Å^2)	g
Na	3d	0.5	0	0	U_{11} = 1.95(8)<br>U_{22} = U_{33} = 3.18(6)	0.987(3)
Te	1b	0.5	0.5	0.5	1.622(5)	1
H	1a	0	0	0	1*	1

[a]Space group 3[illegible] (No. 221), a = 4.76717(5)Å. R_{wp} = 7.07%, GOP = 2.69, R_{Bragg} = 1.14% and R_f = 0.63% for SXRD using JANA2006 program. Rietveld refinement assuming the anion exchange between A and B site ($g_A = g_A$(Te) + g_A(H), $g_B = g_B$(Te) + g_B(H), g_A(H) + g_B(H) = 1, and g_A(Te) + g_B(Te) = 1) yielded R_{wp} = 7.05% with the occupancy of g_A(Te) = 1.0042(5) and g_A(H) = –0.0042(5). The refined composition is $Na_{2.961(9)}HTe$.

*The isotropic displacement parameter is not refined.

Section 5. Electronic band structures at 0 GPa

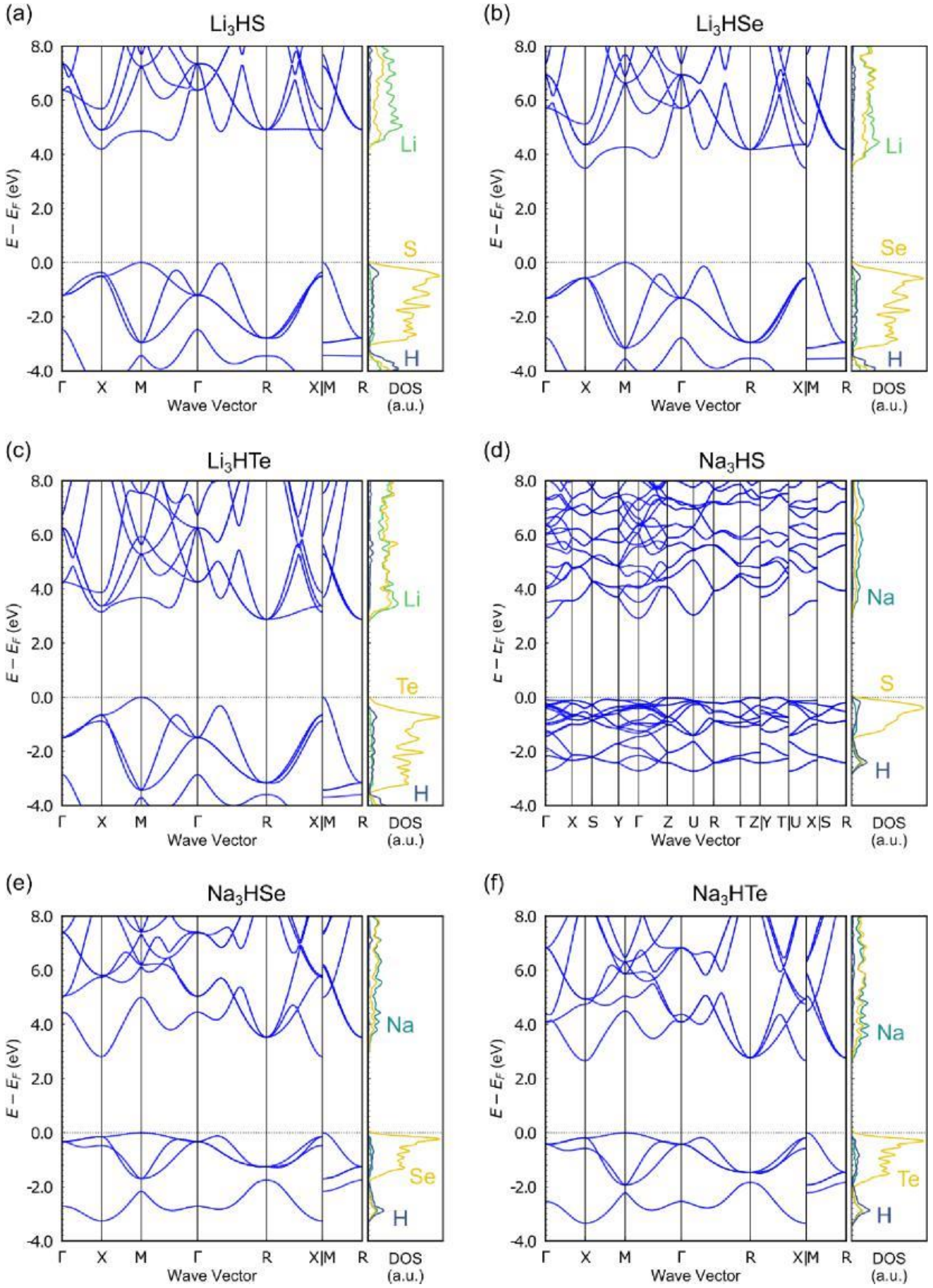

Supplementary Figure 13 Electronic band structures and partial electronic density of states (DOS) of (a) Li_3HS, (b) Li_3HSe, (c) Li_3HTe, (d) *o*-Na_3HS, (e) Na_3HSe, and (f) Na_3HTe under 0 GPa. All plots are normalized by Fermi level, E_F.

Section 6. Phonon band structures at 0 and 5 GPa

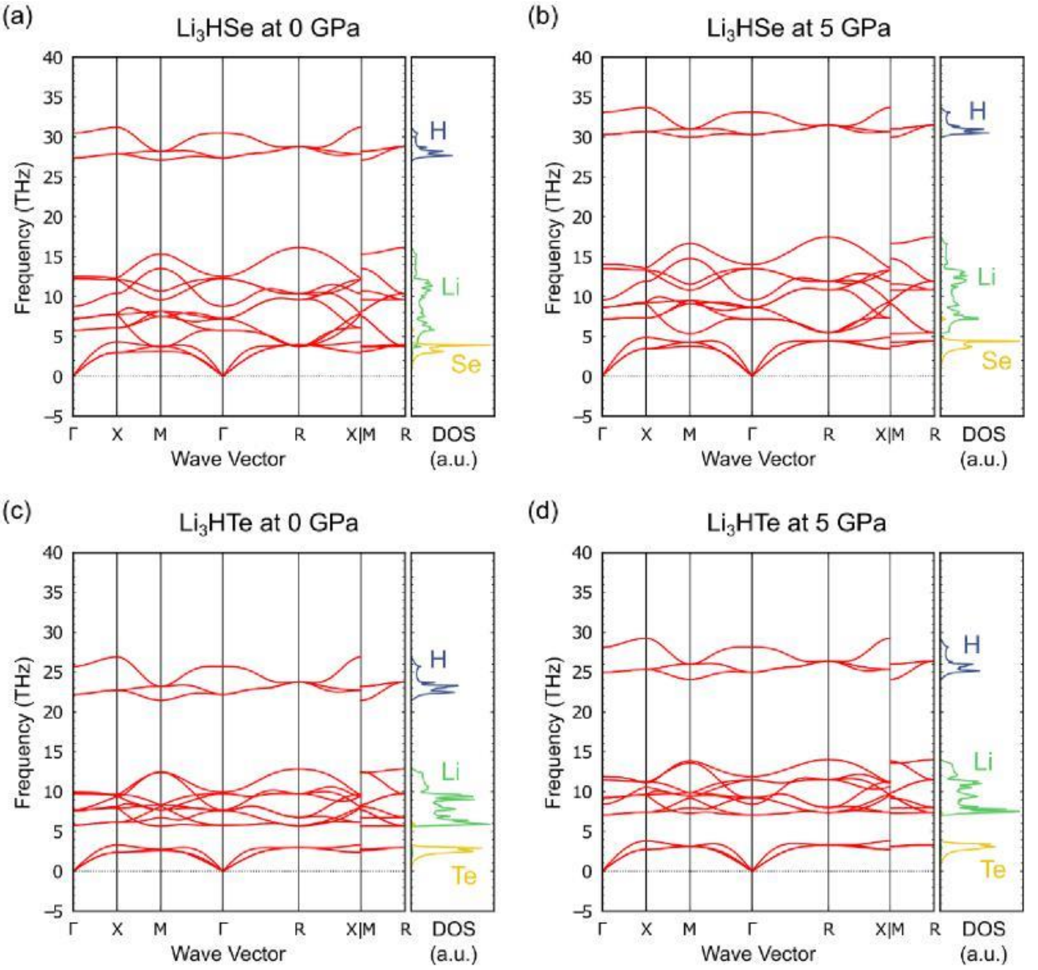

Supplementary Figure 14 Phonon band structures and partial phonon density of states (DOS) of M, H and Ch ions under 0 and 5 GPa. (a,b) Li_3HSe, (c,d) Li_3HTe. Negative number of vertical axis represents imaginary frequency.

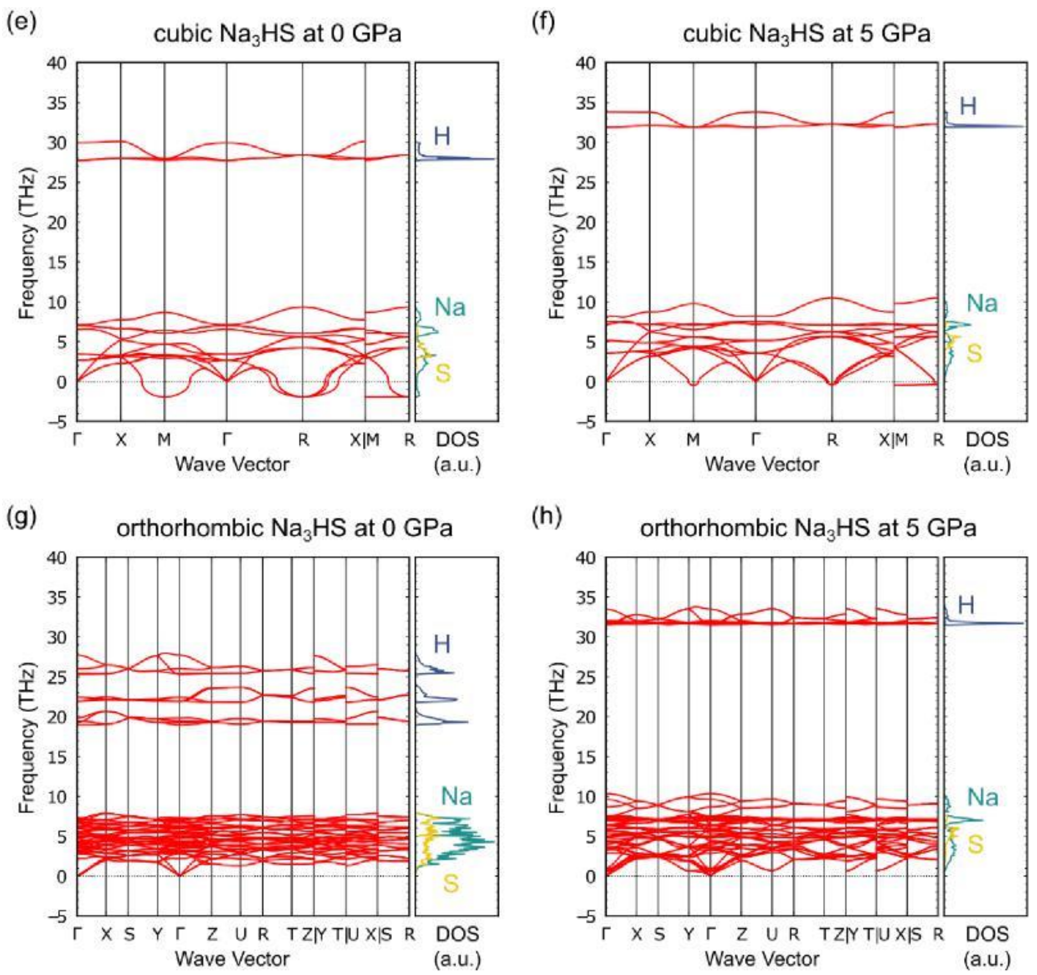

Supplementary Figure 14 (Continued) Phonon band structures and partial phonon density of states (DOS) of M, H and Ch ions under 0 and 5 GPa. (e,f) hypothetical c-Na_3HS, (g,h) o-Na_3HS. Phonon DOS of o-Na_3HS under 5 GPa are similar with that of c-Na_3HS because its crystal structure becomes closer to the cubic form due to the application of pressure. Negative number of vertical axis represents imaginary frequency.

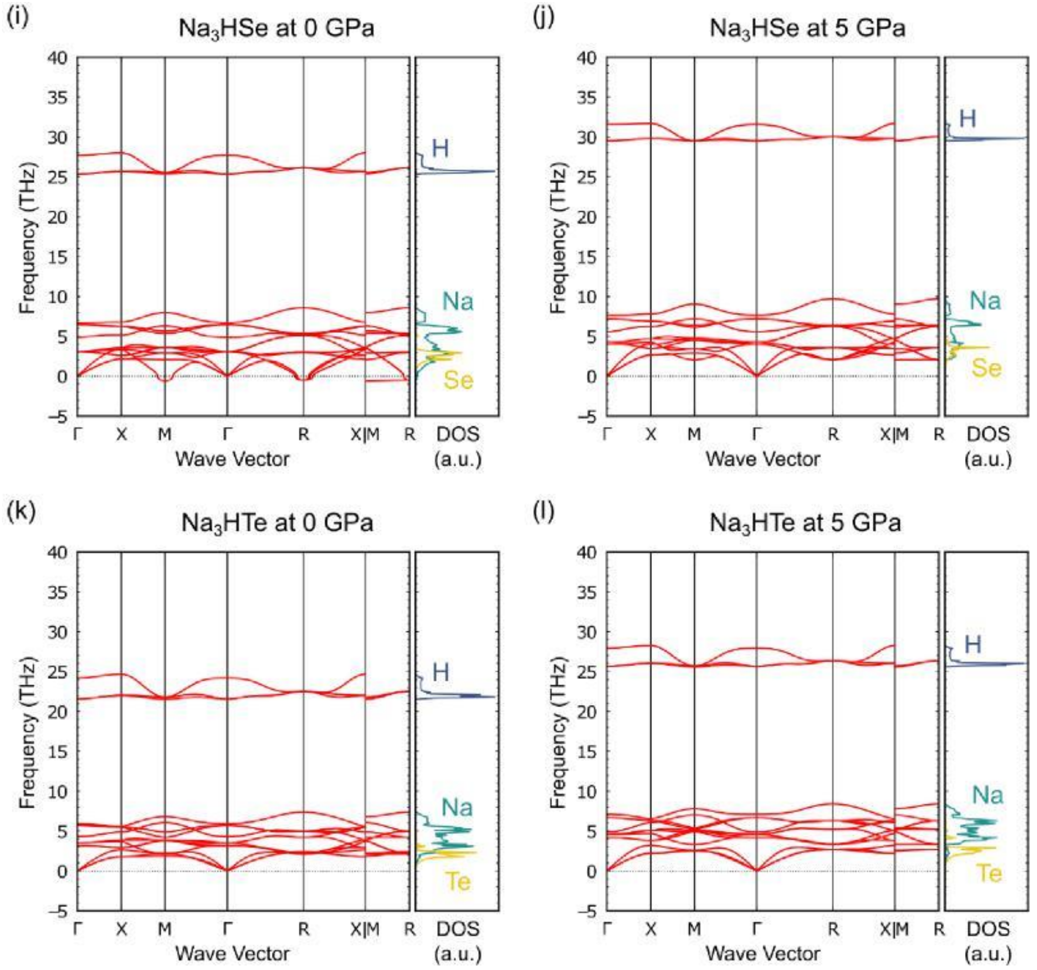

Supplementary Figure 14 (Continued) Phonon band structures and partial phonon density of states (DOS) of M, H and Ch ions under 0 and 5 GPa. (i,j) Na_3HSe and (k,l) Na_3HTe. Negative number of vertical axis represents imaginary frequency.

Section 7. Na^+ migration pathways in orthorhombic Na_3HS

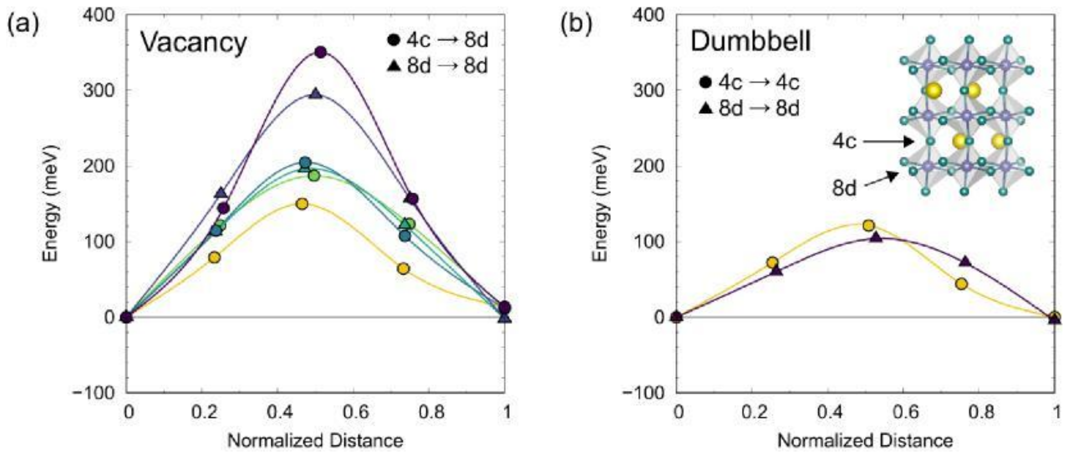

Supplementary Figure 15 Calculated energy migration pathways in orthorhombic Na_3HS via (a) vacancy and (b) interstitial-dumbbell mechanism. The inset in (b) shows Wyckoff positions of Na ions with the crystal structure where light-green, blue and yellow balls represent Na^+, H^-, and S^{2-} ions, respectively. There are six migration pathways for the vacancy mechanism, and the pathway depicted in yellow is displayed in Figure 5 in the main text. The formation energy of Na^+ vacancy at the 4*c* site is slightly lower than that of the 8*d* site by roughly 10 meV. We found only two migration pathways by the dumbbell mechanism, namely from 4*c* to 4*c* and from 8*d* to 8*d* sites, since most of interstitial models that we constructed were not stable and did not fall into local energy minima. The pathway from 4*c* to 4*c* sites is displayed in Figure 5; although the energy is slightly higher than that from 8*d* to 8*d* sites, the formation energy of Na^+ interstitial at the 8*d* site is more stable than that at the 4*c* site by roughly 170 meV.

Section 8. The migration barriers and defect formation energies in M_3HCh

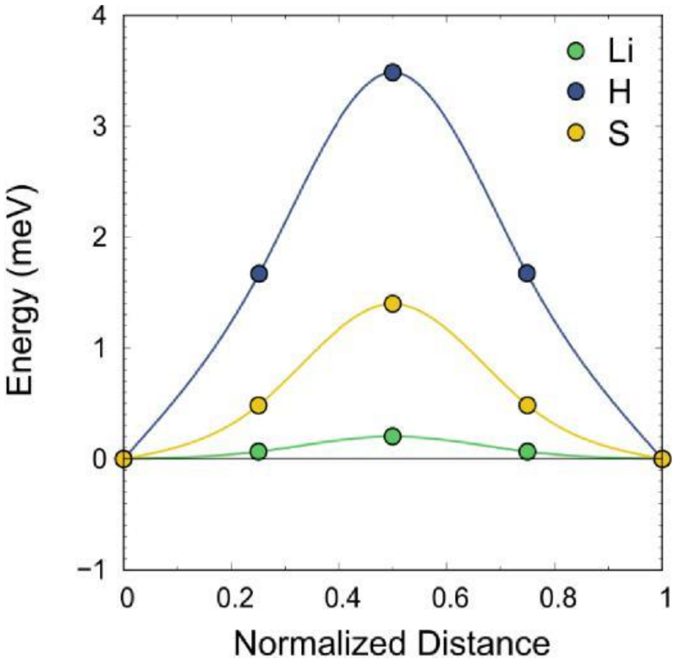

Supplementary Figure 16 Migration energy barriers via vacancy mechanism in Li_3HS. The calculated migration barrier is ~0.20 eV for Li^{+}, ~3.49 eV for H^{-} and ~1.40 eV for S^{2-}.

Supplementary Table 9 Formation energies of Schottky and Frenkel defects. The possible charge carriers include MH Schottky defects with a M-vacancy (V_M') and H-vacancy ($V_H^{\bullet}$), M_2Te Schottky defects with two V_M' and one Ch-vacancy ($V_{Ch}^{\bullet\bullet}$). Charge-neutral M/M-vacancy Frenkel defect pair is expressed by $V_M' + M_i^{\bullet}$ with the Kröger-Vink notations. For o-Na_3HS, the energies of the most stable vacancy and interstitial were used for the calculation. Energies are in eV per defect.

Compound	$V_M' + V_H^{\bullet}$	$2V_M' + V_{Ch}^{\bullet\bullet}$	$V_M' + M_i^{\bullet}$
Li_3HS	0.707	0.653	0.862
Li_3HSe	0.693	0.760	1.016
Li_3HTe	0.542	0.855	1.069
Na_3HS	0.667	0.866	0.893
Na_3HSe	0.647	0.570	0.804
Na_3HTe	0.672	0.767	1.061

Section 9. Ionic diffusion

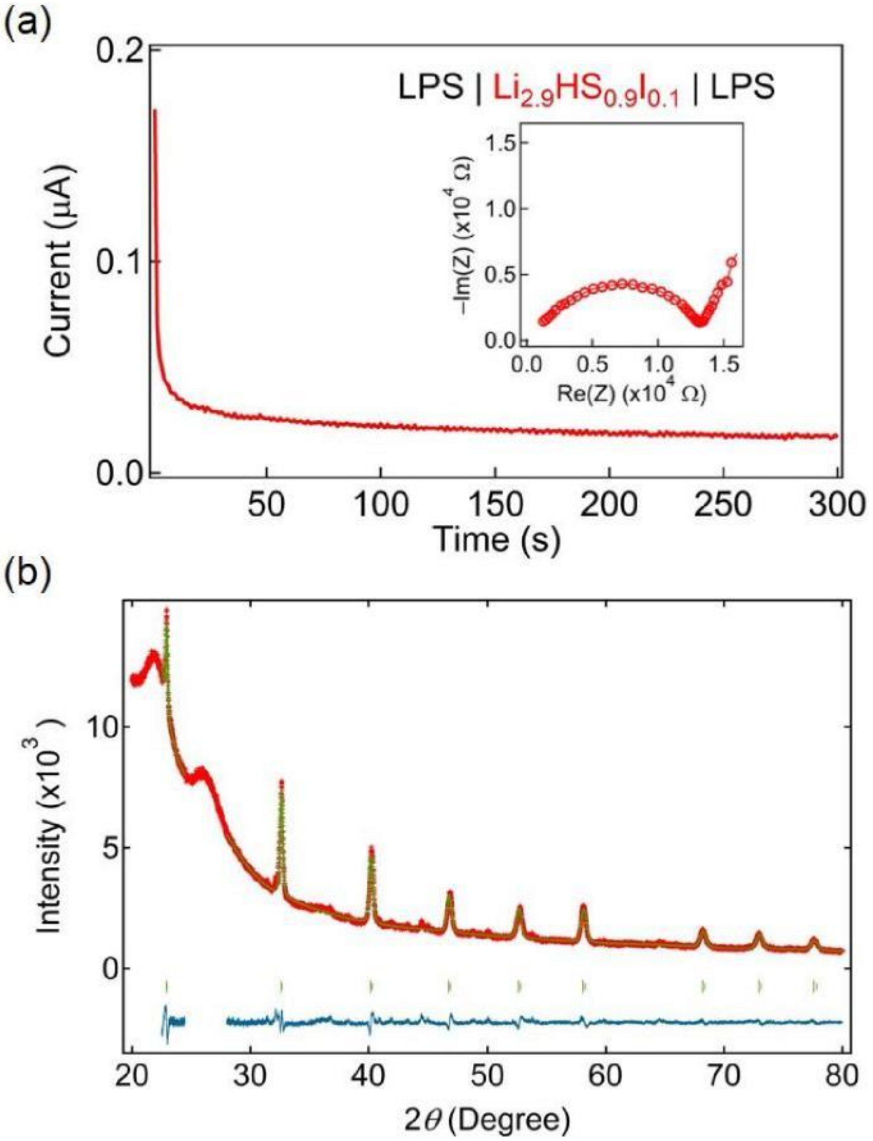

Supplementary Figure 17 (a) The results of potentiostatic measurement of the symmetric cell Li_3PS_4/$Li_{3-x}H(S_{1-x}I_x)$/Li_3PS_4, where Li_3PS_4 is a well-known Li-ion conductor and synthesized by mechanical milling according to the reference (Hayashi, A., Hama, S., Morimoto, H., *et al. J. Am. Ceram. Soc.* **2001,** 84, 477-479). The steady current is observed when a DC voltage of 0.5 V is applied at room temperature. Inset shows the Nyquist plot of the cell Li_3PS_4/$Li_{2.9}H(S_{0.9}I_{0.1})$/$Li_3PS_4$ before the potentiostatic measurement. (b) Le bail analysis of Cu-Kα XRD pattern of iodine doped $Li_{2.9}H(S_{0.9}I_{0.1})$ yielded a cubic phase with a = 3.89205(12) A , R_{wp} = 3.85%, GOF = 1.64. The red crosses, green solid line, the blue solid line, and green dashes denotes the observed, calculated, difference intensities and calculated Bragg reflections, respectively. The following ranges were excluded from the refinement: 5.0–22.5º and 24.5–28.0º.

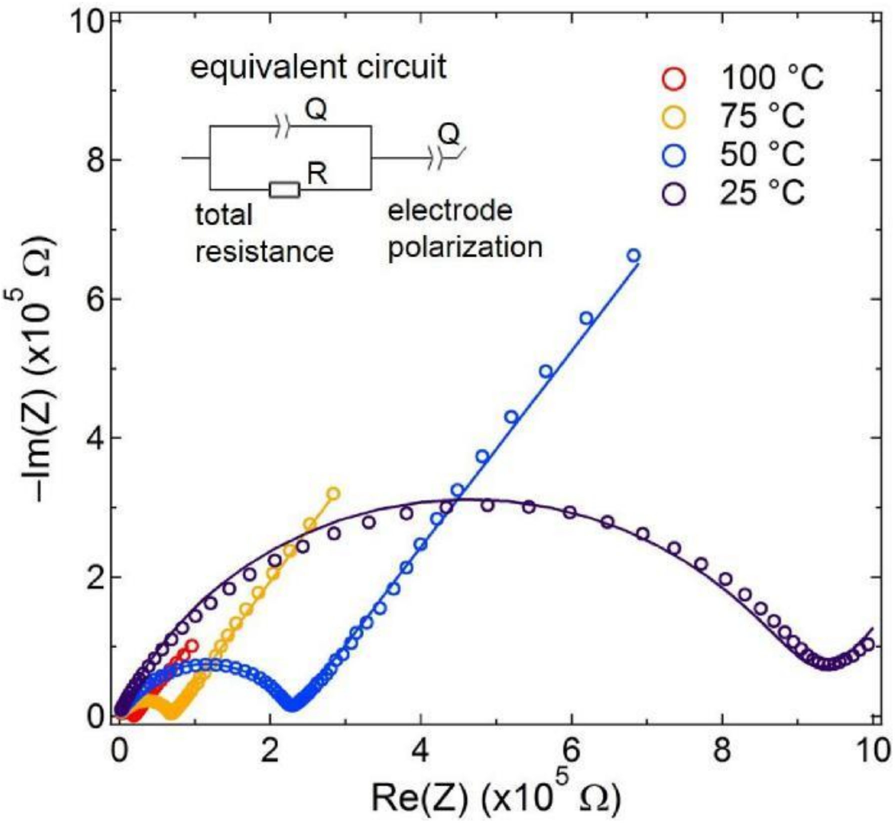

Supplementary Figure 18 Nyquist plots (open circles denote raw data; solid lines denote fitting curves) of cold-pressed Na_3HSe pellet in the temperature range of 25 °C to 100 °C. The spectra were fit with an equivalent circuit consisting of a serial connection of one parallel arrangements of a resistor (R) and a constant phase element (CPE, Q) and an additional CPE component for electrode polarization.

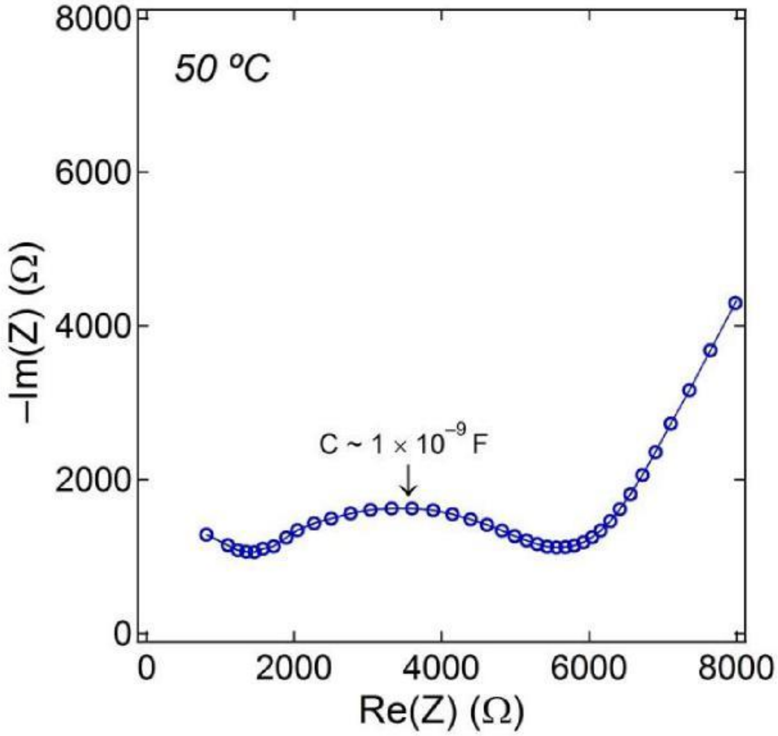

Supplementary Figure 19 Nyquist plots of cold-pressed $Na_{2.9}H(Se_{0.9}I_{0.1})$ pellet at 50 °C. The impedance spectrum at 50 °C features a small half-semicircle in the high-frequency region and a large semicircle from which the apex frequency is ~4.0 □ 10^4 Hz and the corresponding capacity is ~1.0 □ 10^{-9} F. This is a typical value for the grain boundary capacitance of a sample (Irvine, J. T. S., Sinclair, D. C. & West, A. R. *Adv. Mater.* **1990**, 2, 132– 138), and therefore the small half-semicircle with the associated resistance is attributed to the bulk properties of the $Na_{2.9}H(Se_{0.9}I_{0.1})$.

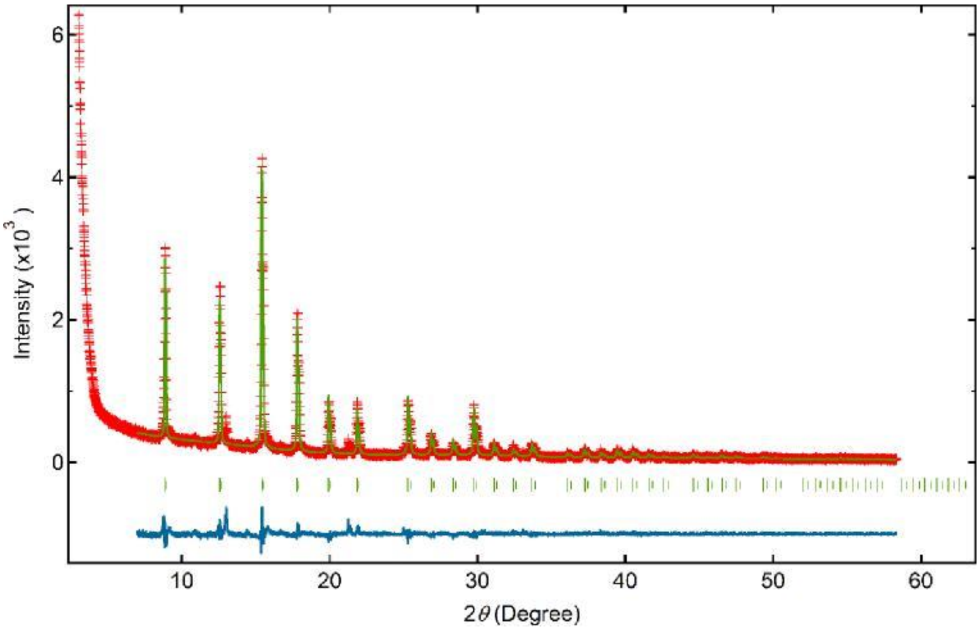

Supplementary Figure 20 Rietveld refinement of XRD (Mo-Kα) pattern of $Na_{2.9}H(Se_{0.9}I_{0.1})$. The red crosses, green solid line, the blue solid line, and green dashes denotes the observed, calculated, difference intensities and calculated Bragg reflections, respectively.

Supplementary Table 10 Atomic coordinates, displacement parameters and occupancy factors for the cubic $Na_{2.9}H(Se_{0.9}I_{0.1})$ using Mo-Kα XRD[a]

Atom	Site	*x*	*y*	*z*	U_{iso}(100 Å^2)	*g*
Na	3*d*	0.5	0	0	4.91(15)	0.964(3)
Se	1*b*	0.5	0.5	0.5	2.67(5)	0.892(8)
I	1*b*	0.5	0.5	0.5	2.67(5)	0.108(8)
H	1*a*	0	0	0	1*	1

[a]Space group *3*□ (No. 221), *a* = 4.57627(16) Å. R_{wp} = 11.84%, GOP = 1.57, R_{Bragg} =5.17% and R_f =3.89% for XRD using JANA2006 program. In order to preserve the charge neutrality of the structure, the refinement was constrained using the equation: 3*g*(Na) = 2*g*(Se) + *g*(I) + *g*(H). The refined composition is $Na_{2.892(9)}HSe_{0.892}I_{0.108}$.

*The isotropic displacement parameter is not refined.

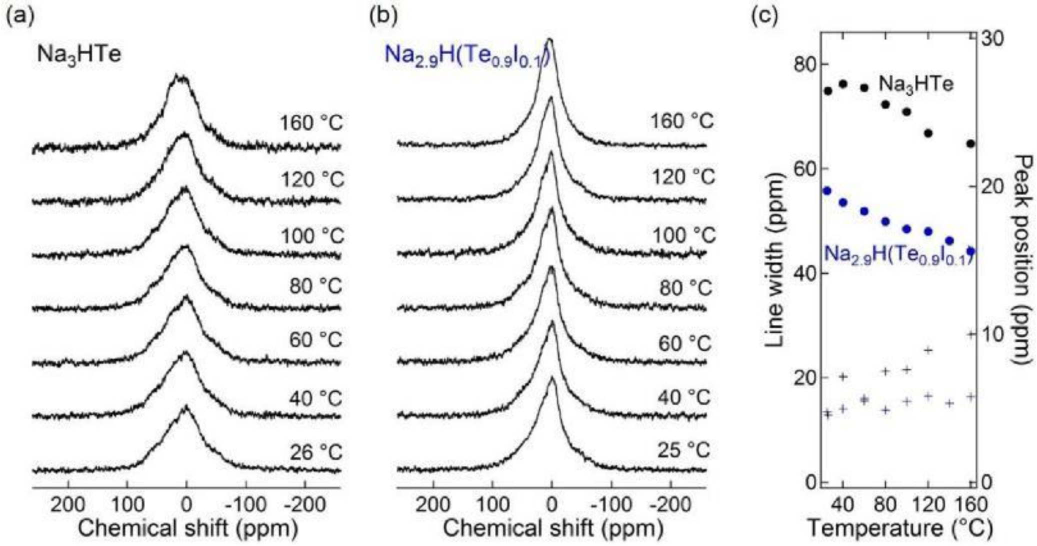

Supplementary Figure 21 Temperature-variable ^{23}Na static NMR spectra of (a) pristine Na_3HTe and (b) $Na_{2.9}H(Te_{0.9}I_{0.1})$ recorded under 7 T magnetic field. (c) Temperature dependence of their line width (circle) and peak position (cross).

Section 10. Supplementary references

S1. Chern, M. Y.; Vennos, D. A.; Disalvo, F. J. Synthesis, Structure, and Properties of Anti-Perovskite Nitrides Ca_3MN, M=P, As, Sb, Bi, Ge, Sn, and Pb. *J. Solid State Chem.* **1992**, *96*, 415–425.

S2. Widera, A.; Schäfer, H. Übergangsformen Zwischen Zintlphasen Und Echten Salzen: Die Verbindungen A_3BO (MIT A = Ca, Sr, Ba Und B = Sn, Pb). *Mater. Res. Bull.* **1980**, *15*, 1805–1809.

S3. Velden, A.; Jansen, M. Zur Kenntnis Der Inversen Perowskite M_3TO (M = Ca, Sr, Yb; T = Si, Ge, Sn, Pb). *Zeitschrift fur Anorg. und Allg. Chemie* **2004**, *630*, 234–238.

S4. Quilty, C. D.; Avdeev, M.; Driskell, J. D.; Sullivan, E. Structural Characterization and Photoluminescence in the Rare Earth-Free Oxy-Fluoride Anti-Perovskites $Sr_{3-x}Bi_{2x/3}AlO_4F$ and $Sr_{3-x}Bi_{2x/3}GaO_4F$. *Dalt. Trans.* **2017**, *46*, 4055–4065.

S5. Prado-Gonjal, J.; Schmidt, R.; Romero, J. J.; Ávila, D.; Amador, U.; Morán, E. Microwave-Assisted Synthesis, Microstructure, and Physical Properties of Rare-Earth Chromites. *Inorg. Chem.* **2013**, *52*, 313–320.

S6. Coutinho, P. V.; Cunha, F.; Barrozo, P. Structural, Vibrational and Magnetic Properties of the Orthoferrites $LaFeO_3$ and $YFeO_3$: A Comparative Study. *Solid State Commun.* **2017**, *252*, 59–63.

S7. Pagnier, T.; Charrier-Cougoulic, I.; Ritter, C.; Lucazeau, G. A Neutron Diffraction Study of $BaCe_xZr_{1-x}O_3$. *Eur. Phys. J. Appl. Phys.* **2000**, *9*, 1–9.

S8. Koopmans, H. J. A.; Van De Velde, G. M. H.; Gellings, P. J. Powder Neutron Diffraction Study of the Perovskites $CaTiO_3$ and $CaZrO_3$. *Acta Cryst.* **1983**, *C39*, 1323–1325.
